# Supplementary material for: Nuclear mechano-confinement induces geometry-dependent HP1α condensate alterations
Source: Commun Biol. 2025 Feb 25;8:308. doi: 10.1038/s42003-025-07732-6 (PMC11862009; doi:10.1038/s42003-025-07732-6)
Supplement: Supplementary file 2 — Supplemental Information [file 42003_2025_7732_MOESM2_ESM.pdf]

Supplementary document

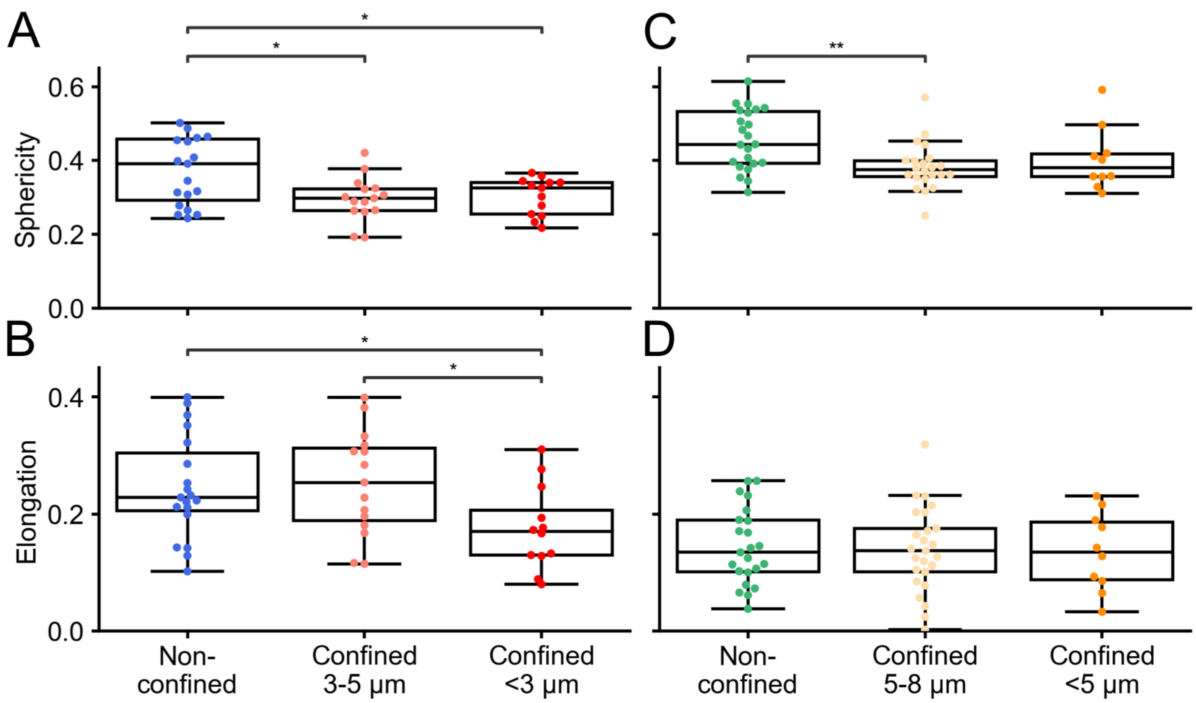

Supplementary Figure 1 Morphological features of sphericity and elongation for nuclei of IMR90 (left column) and HeLa (right column). **A:** Boxplots showing sphericity in non-confined (left box), confined to 3-5 µm (middle box) and below 3 µm (right box) in IMR90 cells (19 in non-confined, 14 in 3-5 µm and 13 in <3 µm, from 6 independent experiments). **B:** Boxplots showing measured elongation of the same nuclei as in panel B (elongation of 1 shows the highest nuclear elongation). **C:** Boxplots showing sphericity in non-confined (left box), confined to 5-8 µm (middle box) and below 5 µm (right box) in HeLa cells (23 in non-confined, 24 in 5-8 µm and 11 in < 5 µm, from 3 independent experiments). **D:** Boxplots showing measured elongation of the same nuclei as in panel C. The boxplots in A-D indicate median (middle line), 25th, 75th percentile (box) and largest and smallest values extending no further than 1.5 x interquartile range (whiskers), except for outliers. *: 1.00e-02 < p <= 5.00e-02, **: 1.00e-03 < p <= 1.00e-02 from Mann-Whitney test two-sided.

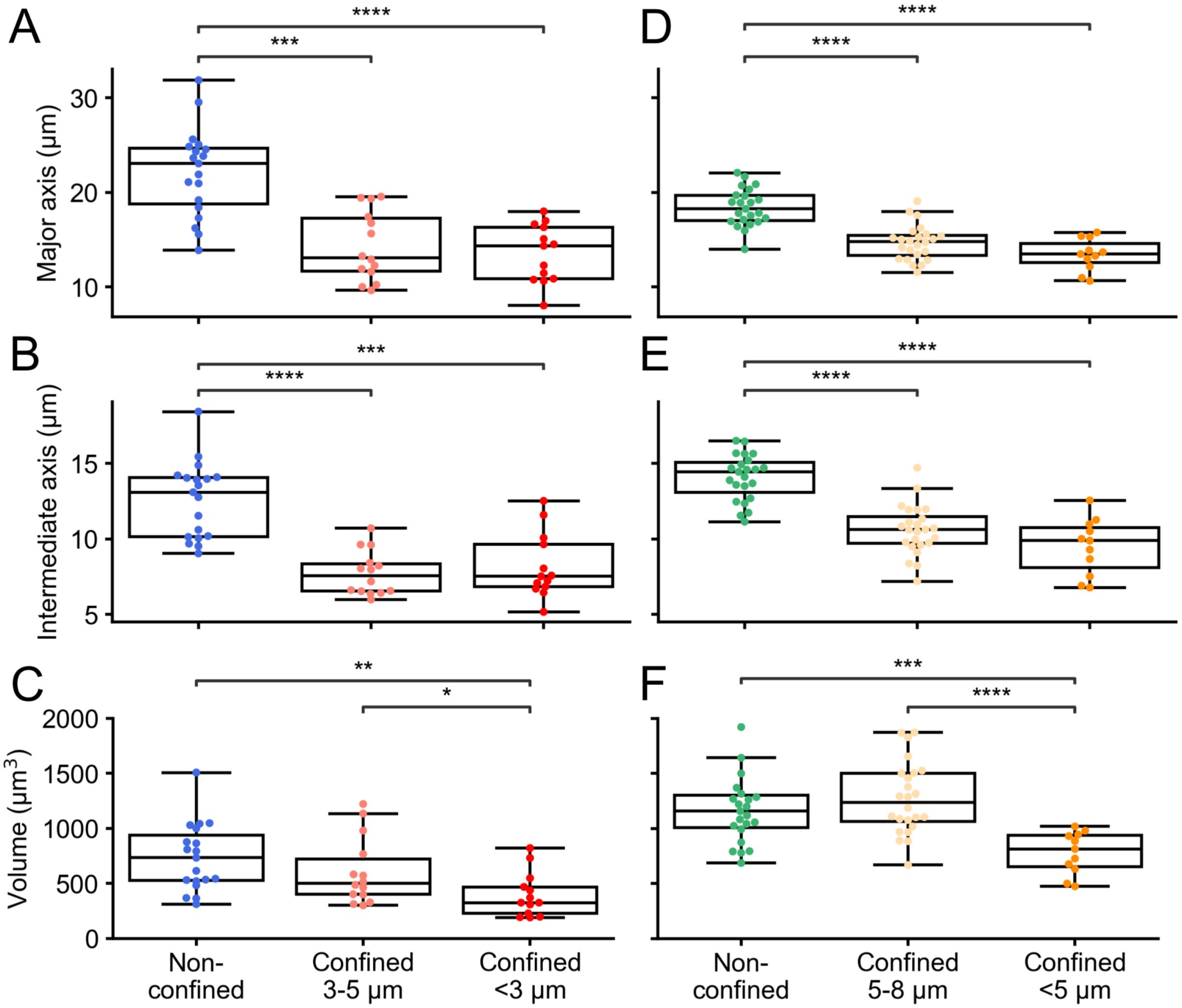

Supplementary Figure 2 Nuclear properties upon cell confinement for IMR90 (left column) and HeLa (right column). **A:** Boxplots showing nuclear major axis diameter (in μm) in non-confined (left box), confined to 3-5 μm (middle box) and below 3 μm (right box) in IMR90 cells (19 in non-confined, 14 in 3-5 μm and 13 in < 3 μm, from 6 independent experiments). **B:** Boxplots showing the measured intermediate axis of the same nuclei as in panel A. **C:** Boxplots showing the measured nuclear volume (in μm^3) of the same nuclei as in panel A and B. **D:** Boxplots showing nuclear major axis diameter (in μm) in non-confined (left box), confined to 5-8 μm (middle box) and below 5 μm (right box) in HeLa cells (23 in non-confined, 24 in 5-8 μm and 11 in <5 μm, from 3 independent experiments). **E:** Boxplots showing the measured intermediate axis of the same nuclei as in panel D. **F:** Boxplots showing the measured nuclear volume (in μm^3) of the same nuclei as in panel D and E. The boxplots in A-F indicate median (middle line), 25th, 75th percentile (box) and largest and smallest values extending no further than 1.5 x interquartile range (whiskers), except for outliers. *: 1.00e-02 < p <= 5.00e-02, **: 1.00e-03 < p <= 1.00e-02, ***: 1.00e-04 < p <= 1.00e-03, ****: p<= 1.00e-04 from Mann-Whitney test two-sided.

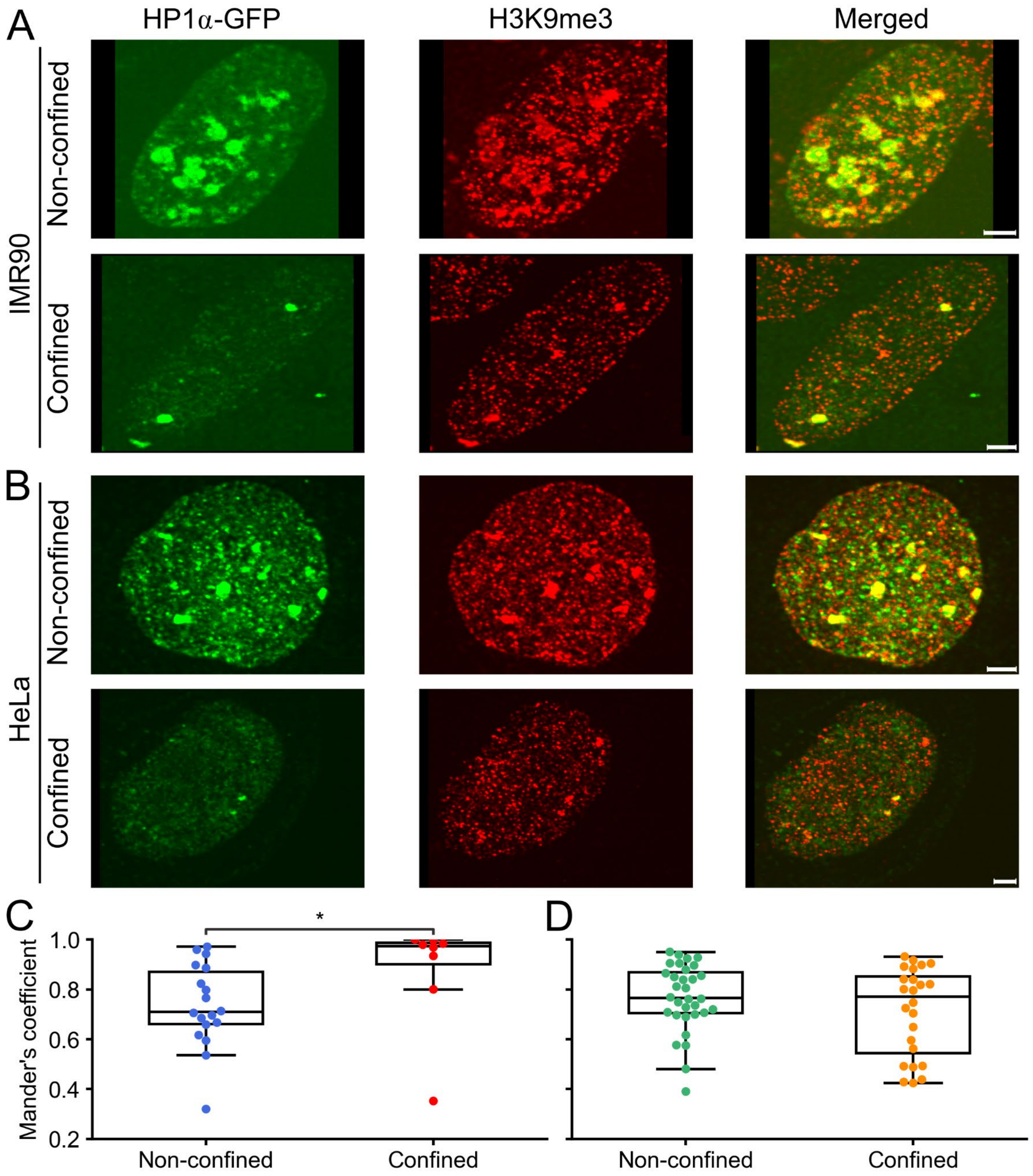

Supplementary Figure 3 HP1α condensates show colocalization with H3K9me3 in IMR90 and HeLa cells in non-confined and confined states. **A:** Super resolution images of nuclei of IMR90 transiently transfected with HP1α-GFP (green), fixed and stained with anti-H3K9me3 (red) antibodies. Merged image shown in the right panel. Scale bar = 2 μm. **B:** Super resolution images of nuclei of HeLa stably transfected with HP1α-GFP (green), fixed and stained with antibody against H3K9me3 (red). Merged image shown in the right panel. Scale bar = 2 μm. **C:** Boxplots showing colocalization analysis of HP1α vs. H3K9me3 with Mander's coefficient to indicate overlap in non-confined (left box; n=258 condensates [in 18 nuclei]) and confined (right box; n=48 [in 9 nuclei]) IMR90 cells from 3 independent experiments. **D:** Boxplots showing colocalization analysis of HP1α vs. H3K9me3 with Mander's

coefficient to indicate overlap in non-confined (left box; n=327 condensates [in 32 nuclei]) and confined (right box; n=181 [in 24 nuclei]) in HeLa cells from 3 independent experiments. The boxplots in C and D indicate median (middle line), 25th, 75th percentile (box) and largest and smallest values extending no further than 1.5 x interquartile range (whiskers). *: 1.00e-02 < p <= 5.00e-02 from Mann-Whitney test two-sided.

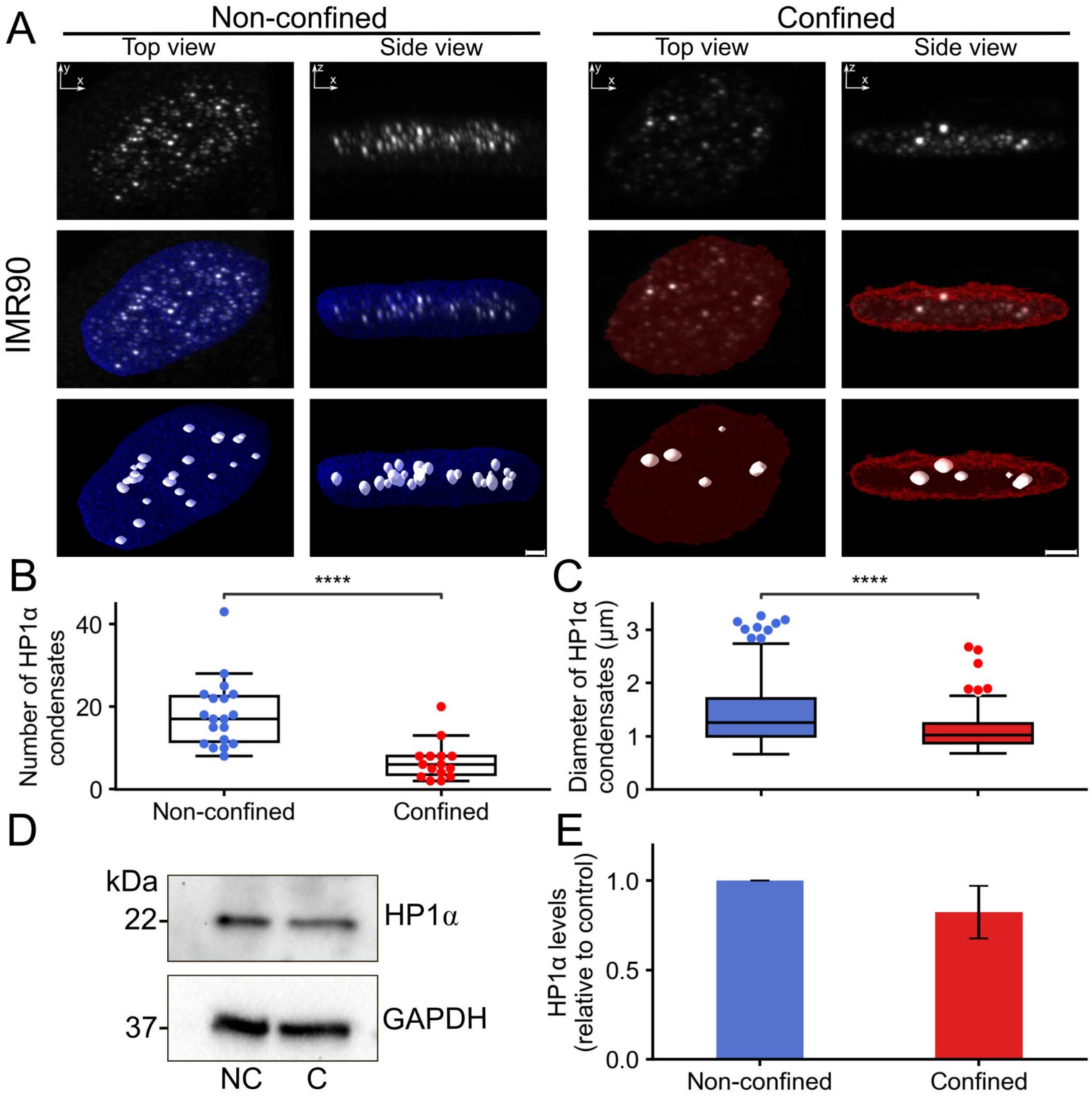

Supplementary Figure 4 Staining of endogenous HP1α condensates in IMR90 shows reduction in number and diameter size, but no change in total protein expression in confined states. **A:** Representative immunofluorescence images of non-confined and confined nuclei with HP1α

condensates of IMR90 fixed and stained with anti-HP1α antibody shown in raw and isosurface top- and side views. Scale bar = 2 μm. **B:** Boxplots showing number of HP1α condensates in IMR90 cells (19 in non-confined and 15 in confined, from 3 independent experiments). **C:** Boxplots showing diameter (in μm) of individual HP1α condensates in non-confined (left box; n=348 condensates [in 19 nuclei]) and confined (right box; n=101 [in 15 nuclei]) in IMR90 cells. The boxplots in B and C indicate median (middle line), 25th, 75th percentile (box) and largest and smallest values extending no further than 1.5 x interquartile range (whiskers), except for outliers. ****: $p \leq 1.00e-04$ from Mann-Whitney test two-sided. **D:** Cell lysates from non-confined (NC) and confined (C) IMR90 were subjected to Western blot analysis with antibodies against HP1α and GAPDH (as a loading control). **E:** Quantification of the intensity of the bands from Western blots with HP1α levels normalized to the amount of GAPDH. Data represents the mean±s.d. relative to the non-confined samples from two independent experiments.

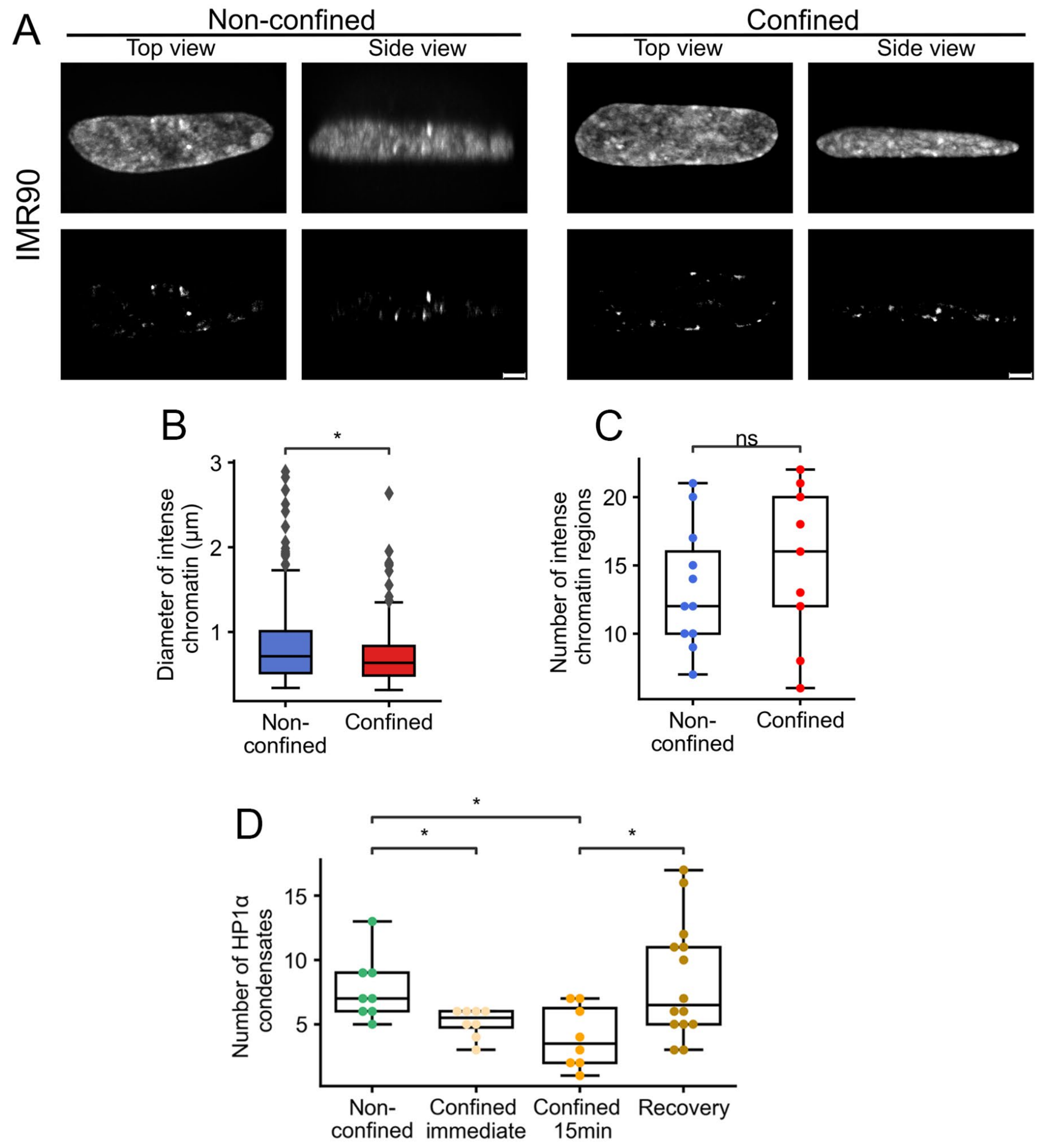

Supplementary Figure 5 DAPI staining of IMR90 cells shows similar numbers and sizes of strong chromatin foci in non-confined and confined states, and the decrease in the number of HP1α condensates is reverted upon confinement release in HeLa cells. **A:** Images of non-confined and confined nuclei for IMR90 cells stained with DAPI in top- and side views. Scale bar: 2 µm. **B:** Boxplots showing diameter sizes (in µm) of individual intense chromatin foci in non-confined (left box; n=147 chromatin foci [in 11 nuclei]) and confined (right box; n=136 [in 9 nuclei]) IMR90 cells. **C:** Boxplots showing number of intense chromatin regions in IMR90 cells (11 nuclei in non-confined and 9 in confined, from 3 independent experiments). **D:** Boxplots showing number of HP1α condensates in non-confined (n=8 nuclei), circa 5 minutes after confinement (confined immediate; n=8 nuclei), after 15 min of confinement (n=8 nuclei) and after removal of the confinement lid (n=14 nuclei) in HeLa cells from one experiment.

The boxplots in B-D indicate median (middle line), 25th, 75th percentile (box) and largest and smallest values extending no further than 1.5 x interquartile range (whiskers), except for outliers. *: 1.00e-02 < p <= 5.00e-02 from Mann-Whitney test two-sided.

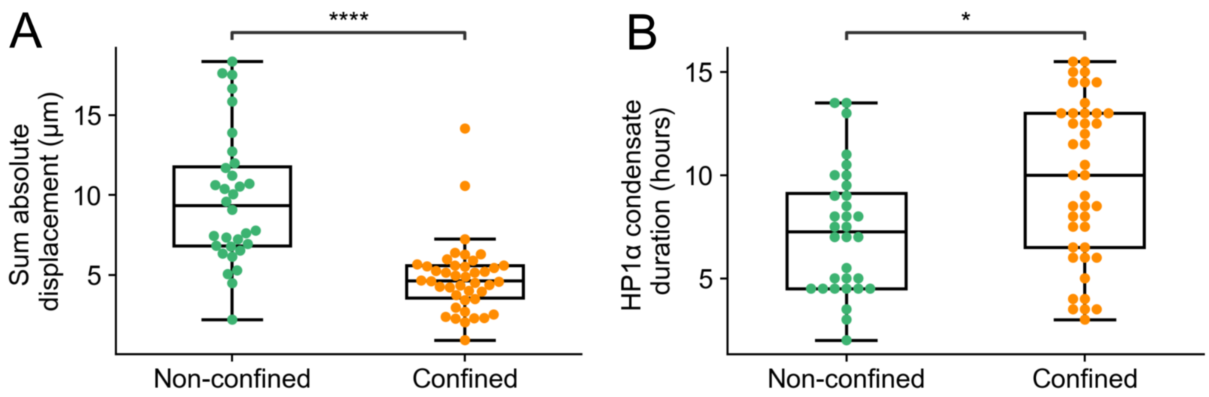

Supplementary Figure 6 HP1α condensates display less absolute displacement and longer durations in confined compared to non-confined states. **A:** Boxplots showing measured sum absolute displacement (μm) for HP1α condensates over 7 hours live-cell videos in non-confined (left box; n=32 condensates [in 9 nuclei]) and confined (right box; n=42 [in 10 nuclei]) HP1α-GFP HeLa cells (from 3 independent experiments). **B:** Boxplots showing total duration in hours for the same HP1α condensates as in A from 15 hour live cell videos. The boxplots indicate median (middle line), 25th, 75th percentile (box) and largest and smallest values extending no further than 1.5 x interquartile range (whiskers), except for outliers. *: 1.00e-02 < p <= 5.00e-02, ****: p<= 1.00e-04 from Mann-Whitney test two-sided.

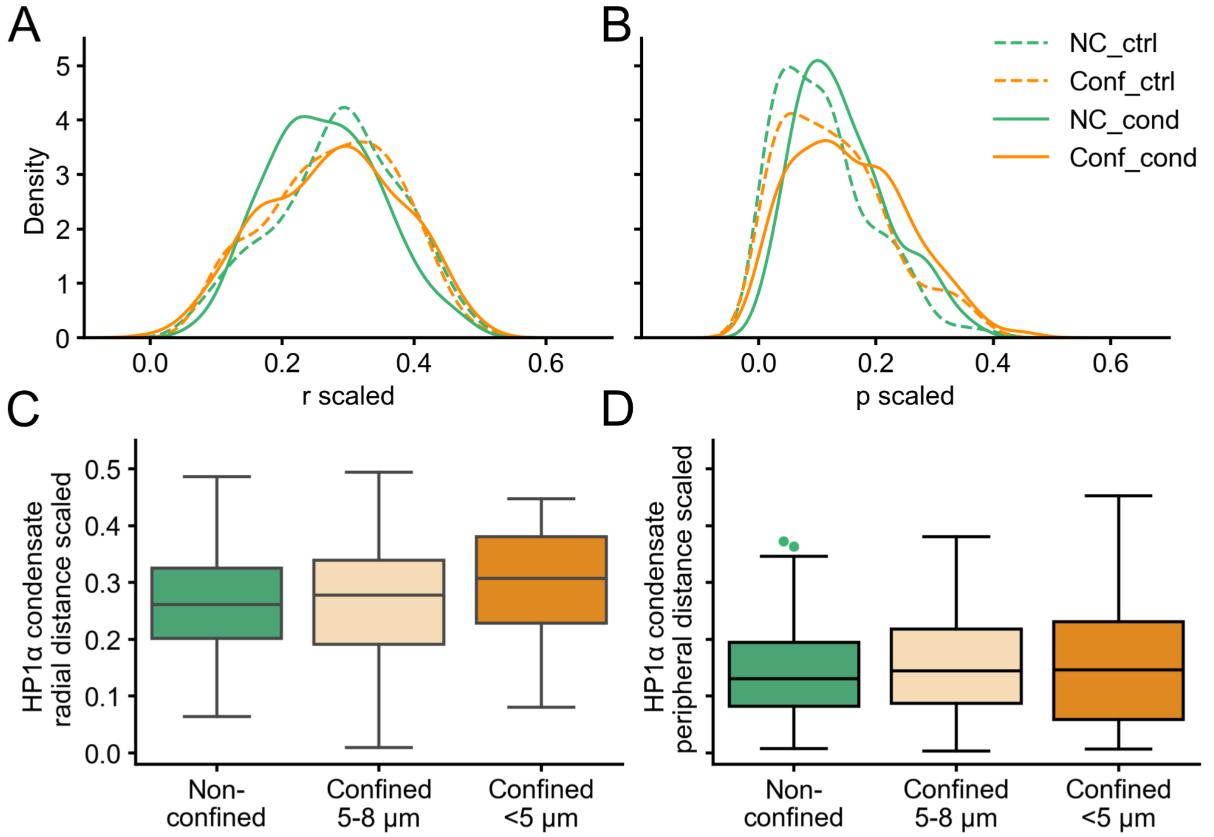

Supplementary Figure 7 HP1α condensates in HeLa cells do not show a shift towards being localized in the center upon confinement. **A:** Kernel Density Estimation (KDE) plots of distributions of radial distances scaled for nucleus diameter of random points (control, dashed lines) and HP1α condensates (smooth lines) in non-confined (green, 224 random points in control and 224 condensates) and confined (orange, 244 random points in control and 244 condensates) cases. **B:** Same as A, but showing nearest distance to the nuclear periphery scaled for nucleus height. **C:** Boxplots of HP1α condensate radial distances scaled for nucleus diameter in HP1α-GFP HeLa cells (left box; n=224 condensates [in 23 nuclei] in non-confined, middle box; n=170 [in 24 nuclei] in 5-8 μm and right box; n=74 [in 11 nuclei] in <5 μm, from 3 independent experiments). **D:** Same as C, but showing the condensate nearest peripheral distances scaled for nucleus height. The boxplots in C and D indicate median (middle line), 25th, 75th percentile (box) and largest and smallest values extending no further than 1.5 x interquartile range (whiskers), except for outliers.

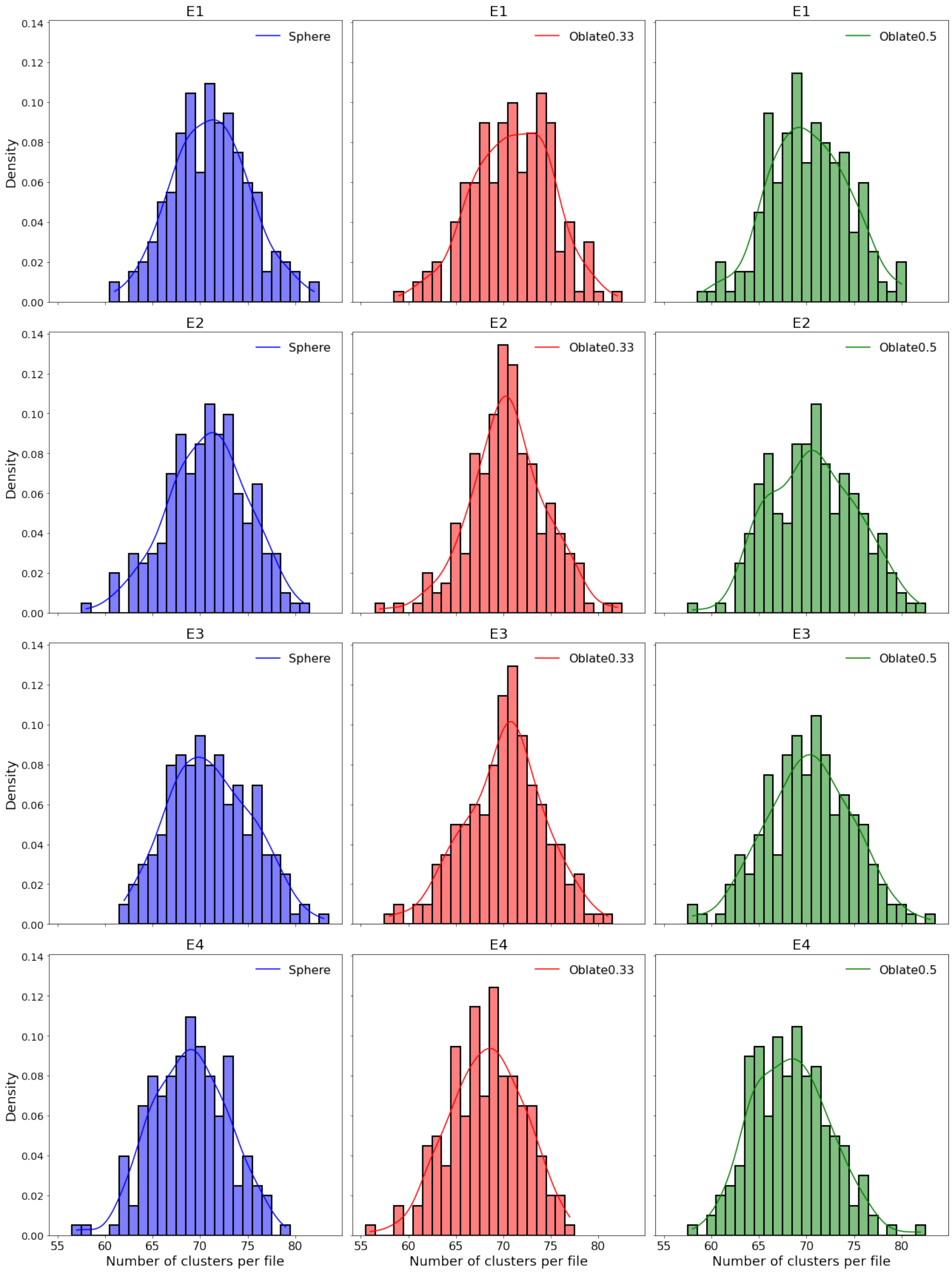
E1
E2
E3
E4
Sphere
Oblate0.33
Oblate0.5
Density
Number of clusters per file
0.00
0.02
0.04
0.06
0.08
0.10
0.12
0.14
55
60
65
70
75
80

Supplementary Figure 8 Distribution of the total number of aggregates per snapshot file from the MD trajectory. The data was collected after the system reached equilibrium and is derived from uncorrelated snapshots. Each row corresponds to a different energy value, ranging from E1 to E4, while each column represents a distinct confinement shape.

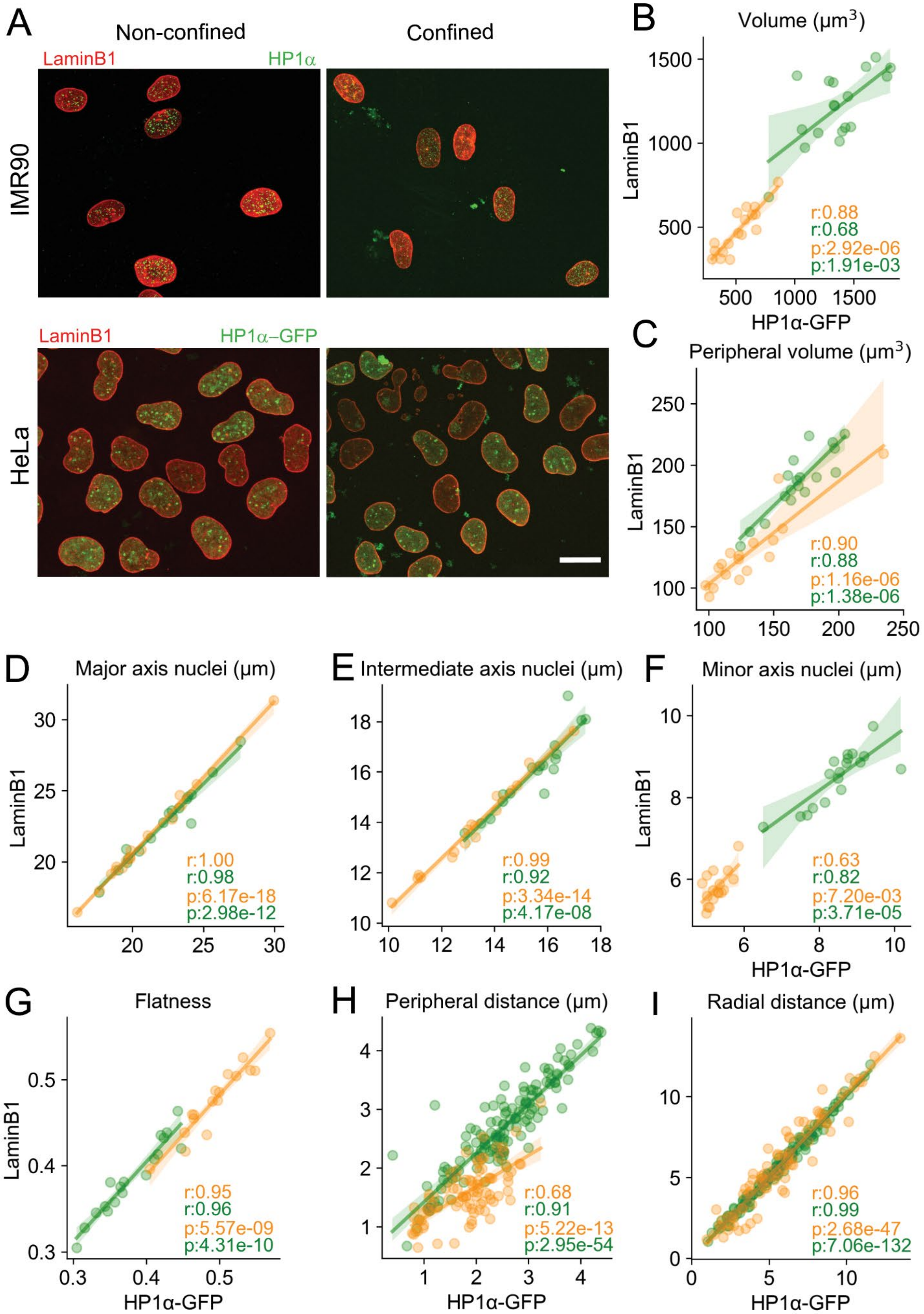
A
Non-confined
Confined
LaminB1
HP1α
IMR90
LaminB1
HP1α–GFP
HeLa
B
Volume (μm^3)
LaminB1
HP1α-GFP
r:0.88
r:0.68
p:2.92e-06
p:1.91e-03
C
Peripheral volume (μm^3)
LaminB1
r:0.90
r:0.88
p:1.16e-06
p:1.38e-06
D
Major axis nuclei (μm)
LaminB1
r:1.00
r:0.98
p:6.17e-18
p:2.98e-12
E
Intermediate axis nuclei (μm)
r:0.99
r:0.92
p:3.34e-14
p:4.17e-08
F
Minor axis nuclei (μm)
LaminB1
HP1α-GFP
r:0.63
r:0.82
p:7.20e-03
p:3.71e-05
G
Flatness
LaminB1
HP1α-GFP
r:0.95
r:0.96
p:5.57e-09
p:4.31e-10
H
Peripheral distance (μm)
HP1α-GFP
r:0.68
r:0.91
p:5.22e-13
p:2.95e-54
I
Radial distance (μm)
HP1α-GFP
r:0.96
r:0.99
p:2.68e-47
p:7.06e-132

Supplementary Figure 9 Correlation analysis between LaminB1 antibody and HP1α-GFP in HeLa shows high correlation across a range of properties. **A:** IMR90 and HP1α-GFP HeLa cells were fixed and stained with LaminB1 antibody (red). IMR90 were also stained with HP1α antibody (green). Representative images of non-confined and confined nuclei (3μm confinement for IMR90 and 5μm confinement for HeLa cells) showing overlapping staining used for correlation quantifications are shown. Scale bar = 20 μm. **B-I:** Structural and morphological parameters in HeLa nuclei between LaminB1 and HP1α-GFP in non-confined (green) and confined (orange) nuclei from one experiment. Pearson's correlation values (r) and p-values (p) are shown in the plots. Peripheral (**B)** and nuclear volume (**C**); Major (**D)**, minor (**E**) and intermediate **(F)** axes diameter for nuclei; Flatness for nuclei (**G);** Peripheral (**H)** and radial (**I)** distances between condensate center to periphery and nucleus center, respectively, for non-confined (141 condensates [in 18 nuclei]) and confined (88 condensates [in 17 nuclei]).
