# Supplementary material for: Nuclear mechano-confinement induces geometry-dependent HP1α condensate alterations
Source: Commun Biol. 2025 Feb 25;8:308. doi: 10.1038/s42003-025-07732-6 (PMC11862009; doi:10.1038/s42003-025-07732-6)
Supplement: Supplementary file 3 — Description of Additional Supplementary File [file 42003_2025_7732_MOESM3_ESM.pdf]

Description of additional supplementary file

File name: Supplementary video

Description: Dynamics of HP1α condensates in HeLa cells stably transfected with HP1α-GFP in non-confined and 5μm confinement states. Representative videos were made from maximum intensity projections of Z-stacks with 0.5 μm step size imaged at 30-minutes intervals for 13 hours. The red circle shows an example of a HP1α condensate undergoing scission and fusion events.
