# Supplementary material for: Nuclear mechano-confinement induces geometry-dependent HP1α condensate alterations
Source: Commun Biol. 2025 Feb 25;8:308. doi: 10.1038/s42003-025-07732-6 (PMC11862009; doi:10.1038/s42003-025-07732-6)
Supplement: Supplementary file 5 — Reporting Summary [file 42003_2025_7732_MOESM5_ESM.pdf]

nature portfolio

Corresponding author(s): Jonas Paulsen and Cinzia Progida

Last updated by author(s): Jan 28, 2025

Reporting Summary

Nature Portfolio wishes to improve the reproducibility of the work that we publish. This form provides structure for consistency and transparency in reporting. For further information on Nature Portfolio policies, see our Editorial Policies and the Editorial Policy Checklist.

Statistics

For all statistical analyses, confirm that the following items are present in the figure legend, table legend, main text, or Methods section.

n/a	Confirmed	
☐	☒	The exact sample size (*n*) for each experimental group/condition, given as a discrete number and unit of measurement
☐	☒	A statement on whether measurements were taken from distinct samples or whether the same sample was measured repeatedly
☐	☒	The statistical test(s) used AND whether they are one- or two-sided<br>*Only common tests should be described solely by name; describe more complex techniques in the Methods section.*
☐	☒	A description of all covariates tested
☐	☒	A description of any assumptions or corrections, such as tests of normality and adjustment for multiple comparisons
☐	☒	A full description of the statistical parameters including central tendency (e.g. means) or other basic estimates (e.g. regression coefficient) AND variation (e.g. standard deviation) or associated estimates of uncertainty (e.g. confidence intervals)
☐	☒	For null hypothesis testing, the test statistic (e.g. *F*, *t*, *r*) with confidence intervals, effect sizes, degrees of freedom and *P* value noted<br>*Give P values as exact values whenever suitable.*
☒	☐	For Bayesian analysis, information on the choice of priors and Markov chain Monte Carlo settings
☒	☐	For hierarchical and complex designs, identification of the appropriate level for tests and full reporting of outcomes
☒	☐	Estimates of effect sizes (e.g. Cohen's *d*, Pearson's *r*), indicating how they were calculated

Our web collection on statistics for biologists contains articles on many of the points above.

Software and code

Policy information about availability of computer code

Data collection	Images aquired with Zeiss LSM880 AiryScan Confocal microscope were processed with Zeiss Airyscan processing function in the ZEN black software.
Data analysis	Image processing and analysis were executed in Python version 3.9 and visualized in the 3D viewer Napari v.0.4.18. Z-stack images were processed with a Gaussian blur filter and segmented by applying an intensity threshold to separate foreground and background voxels to generate a binary image. Nuclei were labeled using connected components labeling while condensates or strong chromatin regions were labeled with eroded-otsu labeling from py-clesperanto v.0.24.1.<br>The Python code used for analyzing the localization of HP1α condensates from polymer simulations is available here: https://doi.org/10.5281/zenodo.10853150<br>Code used to analyze data is available at https://github.com/odasho/mechano-confinement-HP1alpha. Instructions for how to create the software environment used for the analysis are available in the README of our GitHub repository https://doi.org/10.5281/zenodo.14747054. Pinned software versions can be found in the environment.yml file in the GitHub repository.

For manuscripts utilizing custom algorithms or software that are central to the research but not yet described in published literature, software must be made available to editors and reviewers. We strongly encourage code deposition in a community repository (e.g. GitHub). See the Nature Portfolio guidelines for submitting code & software for further information.

Data

Policy information about availability of data

All manuscripts must include a data availability statement. This statement should provide the following information, where applicable:

- Accession codes, unique identifiers, or web links for publicly available datasets
- A description of any restrictions on data availability
- For clinical datasets or third party data, please ensure that the statement adheres to our policy

The dataset for human IMR90 H3K9me3 ChIP-seq data used for polymer simulations can be found in the ENCODE database under the accession number ENCFFF625BTD.

Research involving human participants, their data, or biological material

Policy information about studies with human participants or human data. See also policy information about sex, gender (identity/presentation), and sexual orientation and race, ethnicity and racism.

Reporting on sex and gender	N/A
Reporting on race, ethnicity, or other socially relevant groupings	N/A
Population characteristics	N/A
Recruitment	N/A
Ethics oversight	N/A

Note that full information on the approval of the study protocol must also be provided in the manuscript.

Field-specific reporting

Please select the one below that is the best fit for your research. If you are not sure, read the appropriate sections before making your selection.

☒ Life sciences ☐ Behavioural & social sciences ☐ Ecological, evolutionary & environmental sciences

For a reference copy of the document with all sections, see nature.com/documents/nr-reporting-summary-flat.pdf

Life sciences study design

All studies must disclose on these points even when the disclosure is negative.

Sample size	At least 3 independent experiments were conducted for each conditions. The total number of samples was sufficient from these independent experiments to deduce trends from the data. Statistical differences were assessed using a two-tailed Mann-Whitney test.
Data exclusions	N/A
Replication	Experiments were succesfully replicated at least three times independently
Randomization	N/A
Blinding	Investigators were not blinded as the experiments and quantitative analysis were performed by the same investigator

Reporting for specific materials, systems and methods

We require information from authors about some types of materials, experimental systems and methods used in many studies. Here, indicate whether each material, system or method listed is relevant to your study. If you are not sure if a list item applies to your research, read the appropriate section before selecting a response.

Materials & experimental systems

n/a	Involved in the study
☐	☒ Antibodies
☐	☒ Eukaryotic cell lines
☒	☐ Palaeontology and archaeology
☒	☐ Animals and other organisms
☒	☐ Clinical data
☒	☐ Dual use research of concern
☒	☐ Plants

Methods

n/a	Involved in the study
☒	☐ ChIP-seq
☒	☐ Flow cytometry
☒	☐ MRI-based neuroimaging

Antibodies

Antibodies used

anti-HP1α (Sigma #05-689)
anti-trimethyl-histone H3K9 (Sigma #07-442)
anti-LaminB1 (abcam #ab16048)
anti-GAPDH (SC-47724, Santa-Cruz)

Validation

anti-HP1α (Sigma #05-689):
https://www.sigmaaldrich.com/US/en/product/mm/05689?srsltid=AfmBOoqW9Jcq2xWakkZc5aiuGSuVqKVnQW1AC4bJyO8cJVWuahNFnI9w
From mouse, validated in ChIP, ICC, IHC, IP and WB.

anti-trimethyl-histone H3K9 (Sigma #07-442)
https://www.sigmaaldrich.com/US/en/product/mm/07442?srsltid=AfmBOoqShKIjMRC1gFbU6wB4WtGWCkAzsTHeyTNkSyc3FJr9V8SIOHej
From rabbit, validated in DB, ICC, Mplex, PIA, WB and ChIP-seq.

anti-LaminB1 (abcam #ab16048)
https://www.abcam.com/en-us/products/primary-antibodies/lamin-b1-antibody-nuclear-envelope-marker-ab16048?srsltid=AfmBOoq-Bvh3-daJREUQaBGLK2n7CoXO9UXJkF3GGPBEZ2PTJ4Lcb4d0
From rabbit, KO-validated.

anti-GAPDH (SC-47724, Santa-Cruz)
https://www.scbt.com/p/gapdh-antibody-0411?srsltid=AfmBOopa1i5ZXe10ywgxJc4L_XYK5Eu2ZEEhtg_08aWzLfy1A42WaPJn
From mouse, validated in WB, IP, IF, IHC(P).

Eukaryotic cell lines

Policy information about cell lines and Sex and Gender in Research

Cell line source(s)

IMR90 cells (ATCC)
https://www.atcc.org/products/ccl-186
LOT nr: 64155514

Stably transfected HeLa GFP:HP1α cells (gift from Vagnarelli lab, Brunel University, London)

Authentication

ATCC

Mycoplasma contamination

All cell lines tested negative for mycoplasma contamination.

Commonly misidentified lines
(See ICLAC register)

N/A

Plants

Seed stocks

N/A

Novel plant genotypes

N/A

Authentication

N/A
